# Scalable Hyperpolarized MRI Enabled by Ace‐SABRE of [1‐^13^C]Pyruvate

**DOI:** 10.1002/anie.202501231

**Published:** 2025-07-23

**Authors:** Stephen J. McBride, Megan Pike, Erica Curran, Alexander Zavriyev, Bukola Adebesin, Luke Tucker, Jared M. Harzan, Ishani M. Senanayake, Mustapha Abdulmojeed, Franziska Theiss, Sheng Shen, Thomas Boele, Simon B. Duckett, Boyd M. Goodson, Matthew S. Rosen, Eduard Y. Chekmenev, Hong Yuan, Carlos Dedesma, Terence Gade, Stephen Kadlecek, Thomas Theis, Patrick TomHon

**Affiliations:** ^1^ Department of Chemistry North Carolina State University Raleigh NC USA; ^2^ Department of Radiology University of Pennsylvania Philadelphia PA USA; ^3^ Vizma Life Sciences Chapel Hill NC 27514 USA; ^4^ School of Chemical & Biomolecular Sciences Southern Illinois University Carbondale IL USA; ^5^ A. A. Martinos Center for Biomedical Imaging Massachusetts General Hospital Charlestown MA USA; ^6^ Image X Institute Faculty of Medicine and Health The University of Sydney Sydney NSW Australia; ^7^ Department of Radiology University of North Carolina at Chapel Hill Chapel Hill NC USA; ^8^ Integrative Biosciences, Department of Chemistry, Karmanos Cancer Institute Wayne State University Detroit MI USA; ^9^ Department of Physics North Carolina State University Raleigh NC USA; ^10^ Department of Chemistry University of York York UK

**Keywords:** Hyperpolarization, Metabolic imaging, Magnetic resonance imaging (MRI), Parahydrogen, Pyruvate

## Abstract

Hyperpolarized (HP) MRI using [1–^13^C]pyruvate is emerging as a promising molecular imaging approach. Among hyperpolarization methods, Signal Amplification By Reversible Exchange (SABRE) is attractive because SABRE polarizes the substrates directly in room‐temperature solutions avoiding complex hardware. Most SABRE experiments have historically been performed in methanol, a relatively toxic and difficult‐to‐remove solvent. Here we demonstrate the use of a 80/20 acetone/water (A/W) solvent system (Ace‐SABRE) to provide hyperpolarized [1–^13^C]pyruvate with up to 17% polarization, then implement a solvent processing protocol to achieve injectable solutions retaining 74% of the initial polarization, and lastly we demonstrate HP in vivo spectroscopy and imaging using the Ace‐SABRE platform to showcase metabolic tracking in a hepatocellular carcinoma (HCC) tumor as well as HP‐MRI, both in direct comparison to dissolution dynamic nuclear polarization (d‐DNP) experiments. The Ace‐SABRE technique promises faster adoption of SABRE hyperpolarization in biological experiments, overall lowering the barriers to entry for HP‐NMR and HP‐MRI.

## Graphical abstract

Ace‐SABRE overcomes previous biocompatibility challenges associated with the Signal Amplification By Reversible Exchange (SABRE) hyperpolarization technique. The employed acetone–water mixtures greatly simplify separation of the hyperpolarized compound (e.g., pyruvate) from solvents and catalysts delivering a biocompatible injectable. Ace‐SABRE retains high polarization during processing and enables effective metabolic characterization in vivo.

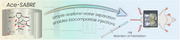

## Introduction

Hyperpolarized (HP) magnetic resonance imaging (MRI) has emerged as an important technology that facilitates noninvasive metabolic imaging by enabling the real‐time study of cellular and molecular processes through its remarkable sensitivity.^[^ ^1^ ^]^ It is the process of hyperpolarization that overcomes the intrinsic limitations in sensitivity of conventional MRI by enhancing nuclear spin polarization levels by several orders of magnitude, which enables the detection of low‐concentration metabolites and the monitoring of their biochemical transformations. One biomarker, [1–^13^C]pyruvate, has proven particularly valuable in this regard due to its central role in metabolism, giving critical in vivo insights into glycolytic and oxidative metabolic pathways in real time.^[^ ^2^, ^3^, ^4^, ^5^, ^6^ ^]^ These capabilities make hyperpolarized pyruvate a powerful diagnostic tool for probing ailments such as cancer,^[^ ^7^, ^8^ ^]^ cardiovascular disease,^[^ ^9^, ^10^ ^]^ and neurodegenerative disorders.^[^ ^11^, ^12^ ^]^ HP‐MRI realizes this advantage for diagnostic and therapeutic MRI applications because the detected changes in chemical shifts instantaneously report on chemical transformations, thereby enabling the simultaneous tracking of multiple metabolic processes in one measurement. This benefit contrasts with the powerful conventional metabolic imaging techniques, positron emission tomography (PET) and single‐photon emission computed tomography (SPECT), whose gamma‐ray signal does not immediately respond to changes in chemical structure. Since HP‐MRI does not expose patients to ionizing radiation, longitudinal studies with frequent assessments become feasible, which allows for the real‐time observation of disease progression or response to treatment. For example, HP pyruvate can distinguish between normal and diseased tissues by encoding changes in metabolic flux, thereby offering critical insights into tumor biology,^[^ ^7^, ^8^ ^]^ neurodegenerative pathways,^[^ ^11^, ^12^ ^]^ or ischemic injuries.^[^ ^13^, ^14^, ^15^, ^16^ ^]^ Unlocking these benefits in a cost‐effective way is critical to improving our understanding of disease mechanisms and enhancing patient outcomes through efficient treatment.

Currently, dissolution dynamic nuclear polarization (d‐DNP)^[^ ^17^ ^]^ is the leading technique for the production of HP metabolic contrast agents such as [1–^13^C]pyruvate. In fact, studies using d‐DNP have demonstrated the utility of HP‐MRI in both preclinical and clinical settings by providing high agent polarization levels with minimal excipients in the injectable solutions.^[^ ^1^, ^18^, ^19^ ^]^ These efforts have led to Phases I and II trials focusing on applications in cancer diagnostics and treatment.^[^ ^1^ ^]^ However, successful d‐DNP faces potential challenges such as a high cost, operational complexity, and lengthy hyperpolarization build‐up time (>60 min). Addressing these challenges improves accessibility and scalability in order to enable high‐throughput commercial clinical HP‐MRI assessments.^[^ ^1^ ^]^ Removing these challenges also allows for more frequent repeated measurements to monitor treatment progression, or disease dynamics, during time‐critical treatment regiments.

To address the current limitations of d‐DNP and access new applications while improving the scalability of HP‐MRI, an alternative pathway to the hyperpolarization of pyruvate and other molecules has been examined. This involves the use of readily formed parahydrogen and, in its broadest sense, has been termed parahydrogen‐induced polarization (PHIP). One approach is the side‐arm hydrogenation (PHIP‐SAH) method, which is already showing significant promise in preclinical studies by achieving sufficient polarization levels for in vivo metabolic imaging with low excipient levels in the injectables.^[^ ^20^, ^21^, ^22^, ^23^, ^24^, ^25^, ^26^, ^27^, ^28^, ^29^ ^]^ The PHIP‐SAH work has demonstrated the value of PHIP‐based methods in characterizing metabolism for high‐throughput applications. A potential difficulty associated with PHIP‐SAH is the need for molecular precursors to pyruvate that are challenging to synthesize and store.^[^ ^24^ ^]^ The PHIP‐SAH approach involves both chemical and physical transformations stemming from the hydrogenation of these precursors, followed by hydrolysis of the product and further purification.^[^ ^20^, ^21^, ^22^, ^23^, ^24^, ^25^, ^26^, ^27^, ^28^, ^29^ ^]^ The benefits of this method, though, are clear, as HP‐pyruvate can be created in minutes meaning there are significant opportunities for future broad dissemination in the healthcare community.

Chemically benign Signal Amplification By Reversible Exchange (SABRE) is emerging as a simpler, more accessible parahydrogen‐based hyperpolarization technique.^[^ ^30^ ^]^ In contrast to PHIP‐SAH, SABRE transfers hyperpolarization directly from parahydrogen into the target molecule via a metal complex whose role is simply to bring parahydrogen and target into contact. SABRE therefore eliminates the need for any complex precursor synthesis and acts quickly because there is no need for a chemical transformation.^[^ ^30^, ^31^, ^32^, ^33^, ^34^, ^35^, ^36^, ^37^ ^]^ Ultimately, these advantages are likely to translate into lower costs and greater ease of adoption. However, the early implementations of SABRE faced challenges, including lower hyperpolarization levels and the use of toxic solvents such as methanol. Recent work has been shown to circumvent some of these challenges by unlocking the first preclinical demonstrations of SABRE‐based HP‐MRI.^[^ ^29^, ^38^, ^39^, ^40^ ^]^ One of the key steps was the implementation of rapid methanol gas stripping; however, small amounts of methanol in the injected solutions remained unavoidable in these solutions. Accordingly, there remain challenges in comparison to PHIP‐SAH, which uses less toxic solvent and delivers hyperpolarization levels commensurate with those of early d‐DNP demonstrations.^[^ ^17^ ^]^

The work presented here showcases ongoing advancements in SABRE hyperpolarization that seek to address current challenges and illustrate a route to clinical viability for SABRE. The current work replaces methanol with acetone, delivers up to 17% polarization, demonstrates the generation of a truly biocompatible solution, provides preclinical in vivo data at multiple sites, and for benchmarking purposes details a side‐by‐side comparison to d‐DNP. The use of the acetone/water (A/W) mixture in the hyperpolarization process involves a more biologically tolerable and easier‐to‐remove solvent than methanol, mitigating some of the safety concerns that arise when producing injectable solutions for in vivo imaging and future clinical translation. In the following, we call this approach Ace‐SABRE.

The A/W mixtures used in this work were purified using common purification methods, liquid–liquid extraction (LLE) and gas stripping.^[^ ^41^, ^42^, ^43^ ^]^ Furthermore, the organometallic SABRE catalyst (Ir‐IMes, where IMes = 1,3‐bis(2,4,6‐trimethylphenyl)imidazol‐2‐ylidene) is most readily soluble in nonpolar solvents and alcohols like methanol. Conversely, pyruvate is soluble in polar solvents, like H_2_O and methanol. Traditionally, this has led to the use of methanol as the solvent of choice for SABRE hyperpolarization chemistry, simplifying the formulation of the hyperpolarization mix and subsequent reaction network. However, methanol is not only a difficult solvent to remove, requiring significant heat and gas flow to remove even small quantities,^[^ ^39^, ^44^, ^45^ ^]^ but is also a Class 2 solvent (FDA/ICH Q3C),^[^ ^46^ ^]^ restricted to minimal permissible daily exposure due to its toxicity. Although previous work has demonstrated the use of ethanol to generate SABRE‐polarized pyruvate, the separation of ethanol from water is difficult to complete under the time‐constrained conditions of hyperpolarized sample processing (requiring removal of impurities and optimization of relaxation effects requiring magnetic field, temperature, and material controls).^[^ ^40^, ^47^ ^]^ Thus, the choice of a solvent with similarly low toxicity to ethanol, such as acetone, introduces a simple alternative that can be more easily removed due to its lower polarity and hence higher affinity for extracting solvents, lower boiling point, and high vapor pressure.^[^ ^41^, ^42^ ^]^ We note that very recently the use of acetone/water mixtures for SABRE was also demonstrated by Bondar et al. to polarize [2–^13^C]pyruvate, but polarization levels remained well under 1%.^[^ ^48^ ^]^

In summary, the optimization of Ace‐SABRE enables polarization levels exceeding 17%, demonstrating the effectiveness of mixed solvent systems surpassing critical thresholds required for effective metabolic imaging. The results are achieved with nondeuterated [1–^13^C]pyruvate, further reducing costs by circumventing the need for deuterated derivatives. These innovations are combined to demonstrate preclinical imaging, where hyperpolarized pyruvate was used to track both spatial distribution and metabolic activity characterizing diseased tissue in vivo.

## Results and Discussion

**Figure 1:**
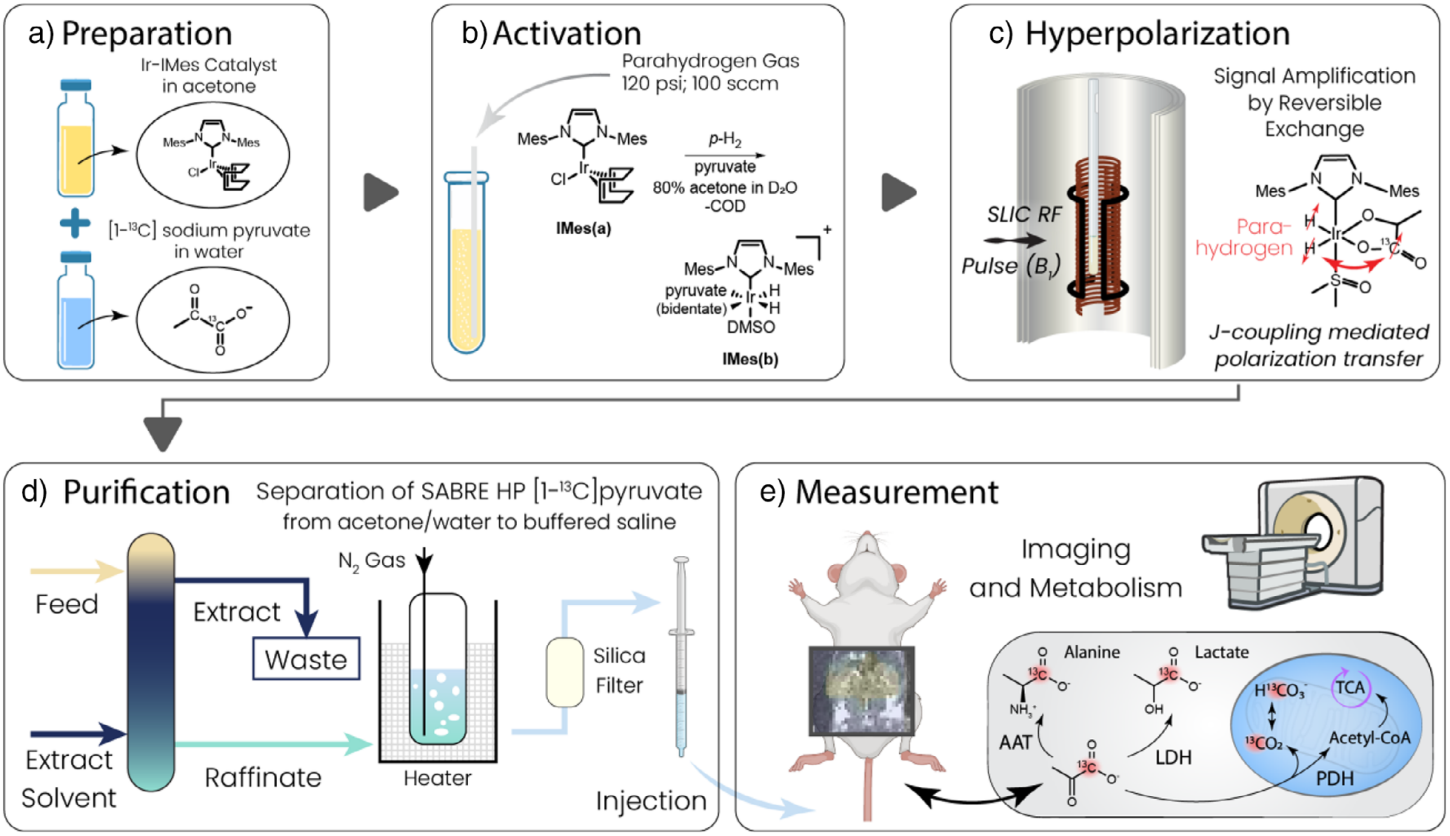
Sample preparation, hyperpolarization, and purification scheme using the present SABRE‐based method. a) Here, an acetone and water (in these experiments, D_2_O) mixture solubilizes the organometallic catalyst and aqueous pyruvate in a homogeneous mixture. b) Introduction of parahydrogen gas hydrogenates the SABRE catalyst precursor to generate a catalytically active system, where c) a pulsed magnetic field controls transfer of the spin order via *J*‐couplings from parahydrogen‐derived hydrides on the SABRE catalyst to target molecules like pyruvate. Reversible exchange on the catalytic center continuously pumps fresh polarization into free pyruvate. d) The HP pyruvate is purified by first extracting the bulk acetone solvent and organic catalyst excipients by liquid–liquid extraction, gas stripping of the remainder of the solvent, and subsequent filtration of remaining iridium with a C18 cartridge filter to render an aqueous solution of HP pyruvate that can be injected into a target subject for imaging.

**Figure 2:**
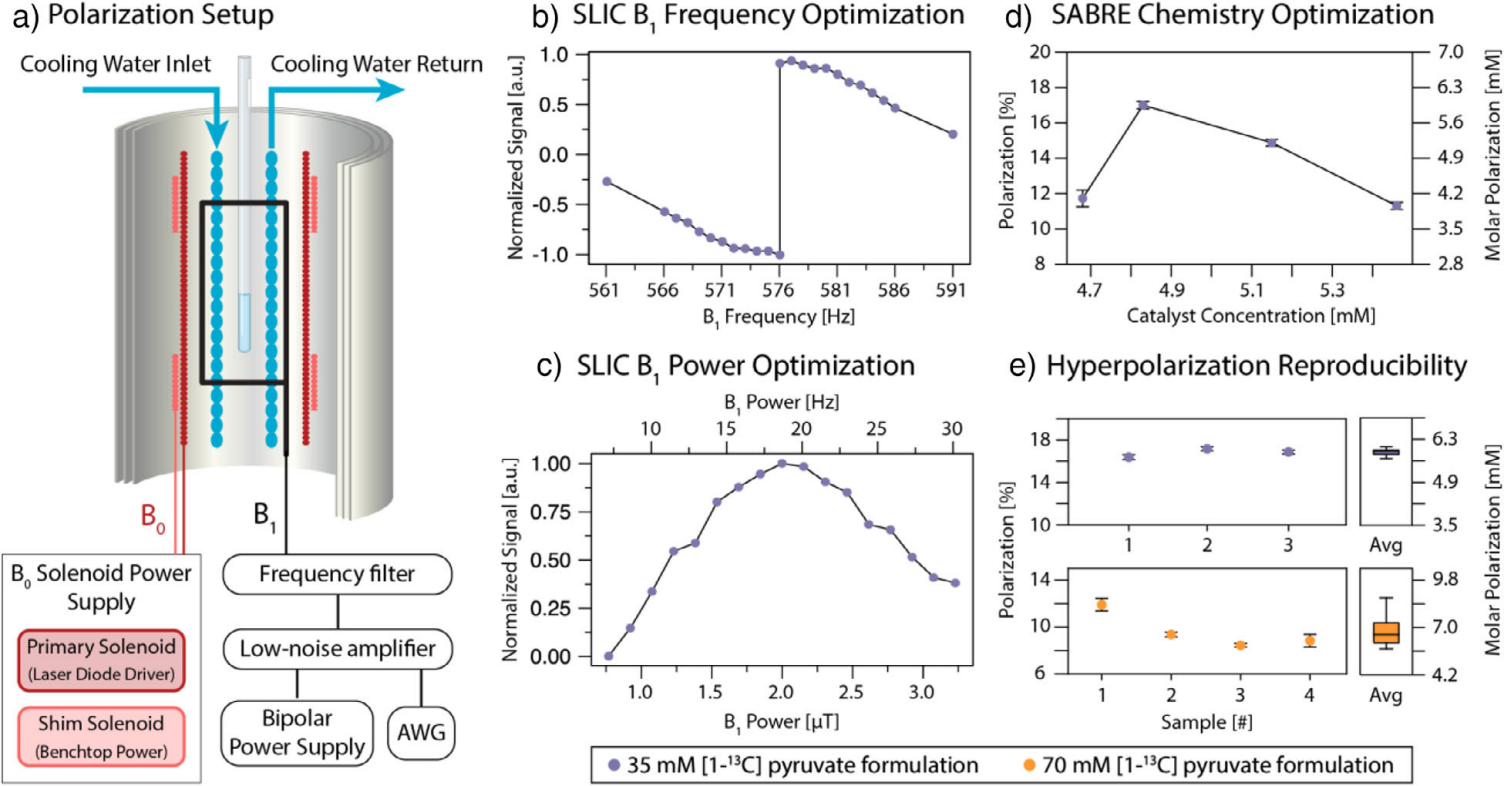
SLIC‐SABRE setup, optimization, and quantification of hyperpolarization. a) SLIC setup operating with a *B*_0_ of 53.8 µT, which is shimmed with the depicted shim solenoid. Water cooling provides sample‐temperature control. b) Hyperpolarized signal as a function of the *B*_1_ frequency. c) Hyperpolarized signal as a function of *B*_1_ amplitude. Note that the applied field is *B*_1_cos(*ωt*) = *B*_1_/2×(e*^iωt ^* + e*^−iωt^*). Thus, only half of the applied field is on resonance. d) Hyperpolarized signal as a function of catalyst concentration at constant DMSO concentration of 12 mM. All samples were prepared from the same catalyst batch with identical conditions and run in sequence on the same day. e) Achieved hyperpolarization noted in % and molar polarization (*p*_m _= polarization × concentration). The temperature of the samples was 6.5 °C for all experiments. In plots B and C, all points were acquired with two separate samples and single scans. In plots D and E, all points represent single samples which were hyperpolarized multiple times to give the corresponding error bars.

In the following results, we show that high polarization (>17%) is achievable on [1–^13^C]pyruvate hyperpolarized with Ace‐SABRE using optimized chemistry in 80:20 A/W solvent mixtures combined with optimized pulsed SABRE techniques, specifically using spin‐lock‐induced crossing (SLIC).^[^ ^49^, ^50^ ^]^ Figure 1 shows how the A/W dual solvent system is coupled with SABRE hyperpolarization to generate aqueous solutions of HP pyruvate that can be injected, imaged, and measured by MRI. First, the sample is prepared (Figure 1a) by mixing the SABRE pre‐catalyst [IrCl(COD)(IMes)], [1–^13^C]pyruvate, and DMSO in a 80:20 acetone:D_2_O mixture. Next, the sample is activated (Figure 1b) by bubbling (100 sccm) parahydrogen gas through the solution at 120 psi (8.3 bar) for 10 min at 6.5 °C. Subsequently, the [1–^13^C]pyruvate is hyperpolarized by SLIC‐SABRE (Figure 1c), and HP‐pyruvate ^13^C polarizations of up to 17.3% are obtained in acetone/water (Figure 2d).

After the hyperpolarization step, the solution is purified using the standard chemical purification and processing techniques of liquid–liquid extraction (LLE) and gas stripping and filtration (Figure 1d). Finally, the in vivo measurements are performed (Figure 1e). The details of these purification, processing, and exemplary in vivo imaging measurements are further detailed below.

### Optimization of SABRE in Acetone/Water

In a 35 mM [1–^13^C]pyruvate SABRE solution, using acetone/water as a solvent, polarization of 16.8 ± 0.4% is achieved before extraction and processing, with 17.3% polarization observed in the best case (Figure 2d,e). For hyperpolarization of these solutions, a mixture of 80:20 acetone to D_2_O is used to dissolve a ratio of 6:1 pyruvate to Ir‐IMes SABRE catalyst (i.e., 35 mM [1–^13^C]pyruvate, 6 mM catalyst). Additionally, DMSO is added as previously described to modulate the exchange rate of the pyruvate.^[^ ^37^, ^51^, ^52^, ^53^ ^]^ In these experiments, a 2:1 ratio of DMSO to catalyst is used (i.e., 12 mM DMSO, 6 mM catalyst). The SABRE precatalyst [IrCl(COD)(IMes)] was prepared as previously described.^[^ ^37^, ^51^ ^]^ The resulting solution is polarized as shown in Figure 2a, where a shimmed *B*_0_ field controls the magnetic field of the sample (∼50 µT), a *B*_1_ saddle coil provides a transverse CW excitation field for spin transfer, and a constantly pumped cooling system provides stable temperature control for the hyperpolarization process.

One of the most important aspects needed to achieve high polarization reproducibly is a strict optimization of the catalyst:DMSO ratio, coupled with optimization of the SLIC pulse parameters (*B*_1_, *B*_0_, and their homogeneity). This is due to the synchronization of both chemical exchange and polarization transfer dynamics in the SABRE hyperpolarization scheme as described previously.^[^ ^54^, ^55^, ^56^, ^57^ ^]^ Because the *J*‐coupling interaction between hydrides and the ^13^C target is relatively weak (∼0.55 Hz),^[^ ^58^, ^59^ ^]^ the exchange rate must be very finely tuned to allow for sufficient polarization flow from hydrides to ^13^C while retaining sufficient catalyst turnover to enable continuous pumping.^[^ ^55^, ^56^, ^57^ ^]^ Similarly, *B*_0_ and *B*_1_ homogeneity are critical within the setup to ensure that all SABRE complexes experience the same conditions and contribute to polarization build‐up irrespective of location in the solution being mixed by the bubbling parahydrogen gas. Figure 2a shows a schematic of our coil configuration, where homogeneity is ensured using magnetic shielding and a shimming solenoid. Figure 2b–d depict separate elements of the multidimensional optimization space, where optimization of the SLIC *B*_1_ frequency, *B*_1_ amplitude, and catalyst concentration at a temperature of 6.5 °C are shown to yield maximization of hyperpolarization on the 1–^13^C site in pyruvate (regarding the *B*_1_ amplitude, we note that the field sensed by the spins in the rotating frame is only half of the plotted applied field because the applied field is *B*_1_ cos(*ωt*) = *B*_1_/2 × (e*^iωt ^* + e*^−iωt^*)). Thus, only half of the applied field is on resonance. In this study, the temperature dimension was not closely examined but relied on prior characterization with SABRE hyperpolarization of [1–^13^C]pyruvate.^[^ ^57^, ^60^ ^]^ Close to on‐resonance, SLIC generates the hyperpolarization aligned with the spin‐locking field in the transverse plane,^[^ ^55^ ^]^ and an adiabatic switch‐off pulse is used at the end of the SLIC period to align x–y magnetization to z‐magnetization as implemented previously.^[^ ^60^ ^]^ In Figure 2b, the *B*_1_ frequency is optimized, including the application of this adiabatic switch‐off pulse.^[^ ^60^ ^]^ This implementation results in a sharp inversion of the peak signal at the resonance frequency of the system at ω_0_ = γ_13C_ *B*_0_ in the *B*_1_ frequency dependence. This is due to production of −z versus +z magnetization above or below resonance. In Figure 2c, the *B*_1_ power is optimized, also including the application of the adiabatic switch‐off pulse. The optimal *B*_1_ power corresponds to a value close to the strength of the *J*_HH_ coupling.^[^ ^55^, ^60^, ^61^ ^]^ Deviations from the exact match can be induced by the exchange dynamics.

We also observe a minimal threshold to the chemical exchange of the system, where below a specific exchange rate driven by the DMSO to catalyst ratio, there is no longer efficient spin transfer due to suppression of exchange and dominating effects of relaxation in the catalytic intermediate (Figure 2d). These results build on past work demonstrating the importance of chemical exchange in modulating the pyruvate and broader alpha‐keto acid SABRE systems,^[^ ^55^, ^56^, ^57^ ^]^ further experimental and theoretical investigation of the broad multiparameter space will be the subject of future studies.

Interestingly, we find no significant difference in achievable hyperpolarization between the [1–^13C^]‐pyruvate and [1–^13^C, 3,3,3‐d_3_]pyruvate isotopomers, which we hypothesize to be due to effects on the *J*‐couplings and active chemical species in the reaction network due to the use of acetone and water as a solvent instead of methanol. Further investigation of the theoretical composition of this reaction network will build on prior work as shown by Lin et al.,^[^ ^62^ ^]^ seeking to deconvolute the differences between these reaction networks.

To obtain more overall signal (i.e., higher molar polarization, *p*_m_), we have reproducibly achieved *p *= 9.7 ± 1.4% (Figure 2e, *n* = 4) on 70 mM pyruvate. On 70 mM [1–^13^C]pyruvate, polarization *p* =  12.5% was achieved in a single experiment. Ratios of DMSO and catalyst for the 70 mM formulation were maintained as above. The variability in these results is due to the stringent chemical and environmental conditions required for hyperpolarization under the reported method, and a fully detailed multiparameter optimization of the 70 mM formulation remains an important future task. These values correspond to *p*_m_ = 9 mM molar polarization (concentration × polarization) for the single (*p *= 12.5%) experiment and *p*_m_ = 7 mM molar polarization for the average (*p *= 9.7%), which is above the molar polarization achieved with the 35 mM formulation (*p*_m_ = 6 mM). Accordingly, we used 70 mM samples for in vivo experiments.

### Pyruvate Processing and Purification

After attaining high Ace‐SABRE polarization purification to biocompatible solution was the next critical task. The first purification step (Figure 1d) follows a traditional LLE scheme,^[^ ^63^, ^64^, ^65^, ^66^ ^]^ where the HP solution of acetone, water, catalyst, and HP [1–^13^C]pyruvate is fed into the extracting solvent butyl acetate. Butyl acetate is chosen due to the low toxicity,^[^ ^46^ ^]^ low water solubility,^[^ ^67^ ^]^ and high affinity of esters for acetone extraction.^[^ ^41^, ^43^ ^]^ The raffinate then consists of the recovered aqueous fraction, containing HP [1‐^13^C]‐pyruvate and residual amounts of acetone, butyl acetate, and iridium catalyst. The goal of the extraction is to maximize the concentration and the polarization of [1–^13^C]pyruvate in the aqueous layer while minimizing the concentrations of acetone and catalyst. In addition to LLE, the raffinate is further purified using gas stripping (nitrogen) at elevated temperature to further reduce the residual acetone. The last purification step is filtration with a C18 silica cartridge filter to further reduce the residual iridium catalyst level. The results of processing a 70 mM SABRE HP [1–^13^C]pyruvate solution to produce a purified solution using these combined purification methods are shown in Table 1. The primary reasons for polarization loss from the original to processed polarization are relaxation effects, which are controlled in the processing by an ∼100 mT permanent magnet array. Importantly, the average polarization observed after the processing steps is *p *= 6.3 ± 0.2%. This corresponds to a retention of 74 ± 3% of the original polarization, which was *p* = 8.5 ± 0.5% in the purification study.

**Table 1:** Resulting concentrations remaining after the purification of SABRE HP, 70 mM [1–^13^C]pyruvate using the method described (*n* = 3). The iridium, acetone, and butyl acetate concentrations are below those of the no observed adverse effect level (NOAEL) for rats using respective FDA/ICH Q3D and Q3C guidelines. Excipient concentrations and pyruvate concentration quantification methods are detailed in the Supporting Information.

Solution output metrics	Concentration or polarization
Iridium	2.7 ± 0.8 µg mL^−1^ (14 ± 4 µM)
Acetone	120 ± 25 mM
Butyl acetate	0.7 ± 0.1 mM
Sodium [1–^13^C]pyruvate before extraction	70 mM
Sodium [1–^13^C]pyruvate after extraction	67 ± 2 mM
Polarization before extraction	8.5 ± 0.5%
Polarization after extraction	6.3 ± 0.2%
Percentage hyperpolarization retained	74 ± 2%

### in vivo Demonstrations

**Figure 3:**
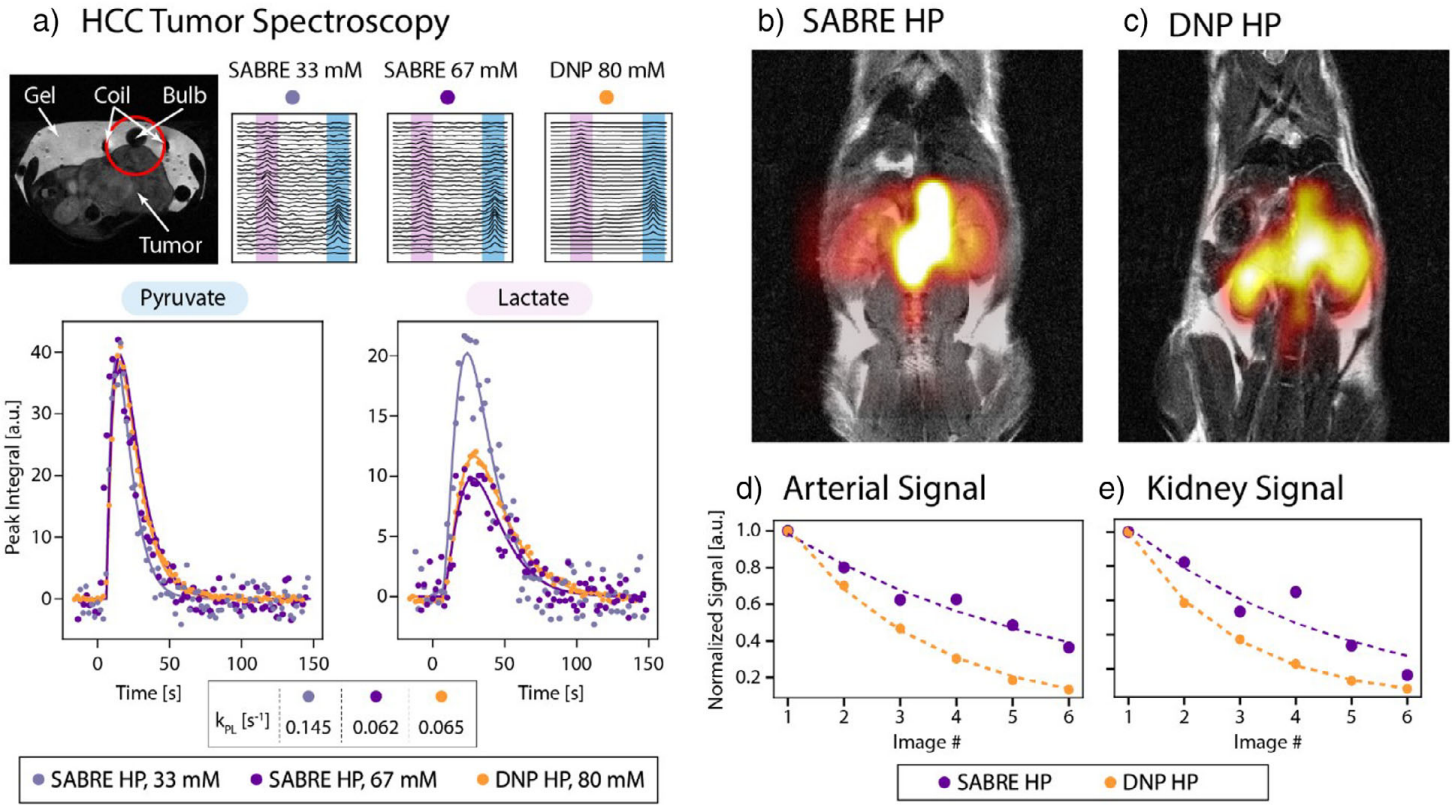
Comparison of results from Ace‐SABRE and d‐DNP injections of hyperpolarized [1–^13^C]pyruvate in both a tumor mouse model (patient‐derived xenograft hepatocellular carcinoma flank tumor) a) and healthy mouse model (BALB/C) b)–e). a) One mouse underwent three subsequent injections with 67 mM Ace‐SABRE HP [1–^13^C]pyruvate, 80 mM d‐DNP HP [1–13C]pyruvate, and 33 mM Ace‐SABRE HP [1–^13^C]pyruvate. a) Top: Labeled ^1^H axial image of the tumor model mouse, depicting the approximate 12 mm surface coil and its sensitive volume. Shown next to the ^1^H image are the surface coil spectroscopy results, with pyruvate peaks highlighted in blue and lactate peaks highlighted in pink. Bottom: Integration of these signals is shown in the plots below, with fitting of both the pyruvate and lactate signal evolution to derive the pyruvate to lactate conversion kinetics (*k*_PL_), shown in the table below the plots. b) EPSI summed image of the pyruvate distribution in a healthy mouse from an injection of 67 mM Ace‐SABRE HP [1–^13^C]pyruvate. c) EPSI summed image of the pyruvate distribution in a healthy mouse from an injection of 80 mM DNP HP [1–^13^C]pyruvate. d) and e) Sum of the arterial voxels (d) and kidney voxels (e) evolving over six acquired EPSI images for Ace‐SABRE HP and DNP HP [1–^13^C]pyruvate. Differences in signal decay are possibly due to varied respiration of the animal and gating of the acquisitions in the respective experiments.

Using these methods, in vivo ^13^C imaging and spectroscopy demonstrate the utility of the technique and enable the results to be compared with established d‐DNP methods. d‐DNP pyruvate doses were prepared with an Oxford Instruments Hypersense polarizer as previously described.^[^ ^68^, ^69^ ^]^ Polarization of [1–^13^C]pyruvate in the d‐DNP produced solutions was ∼20%. All in vivo data was acquired using an MR Solutions 7T cryogen‐free MRI. In Figure 3, we present characterization of tumor biology using Ace‐SABRE HP pyruvate (a) and spectroscopic imaging in a healthy animal (b–e).

Pyruvate to lactate metabolic conversion was evaluated in a tumor‐bearing mouse (patient‐derived xenograft hepatocellular carcinoma flank tumor^[^ ^70^ ^]^) receiving three intravenous HP [1–^13^C]pyruvate injections via the tail vein. Injections were spaced every 30 min, as previously shown to have little impact on the animal's metabolism.^[^ ^71^ ^]^ Following d‐DNP or Ace‐SABRE hyperpolarization, samples were injected (6.7 mL kg^−1^) via central line catheter over a 10‐s period. Using a 12 mm surface coil placed over the tumor (see ^1^H image in Figure 3a, Top), ^13^C spectra were acquired every 2 s for 80 transients using a 15° flip angle. All injections were made in TE (Tris‐EDTA) buffered saline. In Figure 3a, the acquired data is plotted for all three injections with 15 Hz exponential line broadening and analyzed by a custom Python code to calculate the pyruvate to lactate conversion kinetics (*k*_PL_, pyruvate to lactate conversion rate). The observed SNR is lower from the Ace‐SABRE injections due to lower injected polarization level, as well as the lower injected concentrations of pyruvate. However, we show that even with these lower SABRE molar polarizations (*p*_m_ = concentration × polarization), the same *k*_PL_ values are obtained for experiments with a similar injection concentration. These results show the impact on pyruvate transport, LDH activity, or depletion of the NADH pool when varying pyruvate concentrations are injected, as observed before.^[^ ^72^ ^]^ Additionally, we demonstrate that at a lower concentration of pyruvate, we observe an increased *k*_PL_ due to the lower relative saturation of the pyruvate transporters, mirroring previously published literature results.^[^ ^72^, ^73^, ^74^ ^]^

Additionally, we demonstrate imaging of HP pyruvate in a healthy mouse model (BALB/c nude mouse) using both Ace‐SABRE and d‐DNP. Two separate mice were injected with HP pyruvate produced by either Ace‐SABRE or d‐DNP (Figure 3b,c). Following dissolution (d‐DNP, 80 mM HP pyruvate) or our Ace‐SABRE purification (SABRE, 67 mM HP pyruvate), samples were injected (6.7 mL kg^−1^) via tail vein catheter over a 10 s period, and respiratory gated coronal echo‐planar spectroscopic imaging (EPSI) was acquired every 3.5 s, starting 15 s after injection start. Mice imaging was conducted using a volume coil and EPSI with the following parameters: matrix size/resolution/TR/flip angle/slice thickness of 12 × 12/2 × 2 mm/60 ms/5°/6.5–7 mm. The data was analyzed using custom MATLAB code. Anatomic distribution of the pyruvate signal across the arterial and kidney voxels is similar in both the d‐DNP and SABRE injections, but higher relative pyruvate signal in the kidneys is observed with the d‐DNP injection. In separate experiments, we saw similar effects on the biodistribution of the pyruvate in healthy mouse models, implying that the decreased pyruvate signal in the kidneys is due to either lower overall molar polarization of the current SABRE injection or difference in the excipients between the two injections. Additionally, integration of the arterial and kidney voxels in both the SABRE and d‐DNP injections shows similar evolution of both signals during the series of images acquired (Figure 3d,e), with differences in the signal decay possibly due to differences in the respiration and gating of the EPSI acquisitions in the respective experiments.

## Conclusion

This study demonstrates the viability of Ace‐SABRE for preclinical HP‐NMR and HP‐MRI experiments. We believe that Ace‐SABRE will enable the cost‐effective, safe, and scalable production of a hyperpolarized agent while avoiding the use of problematic solvents like methanol or perfluoroalkyl substances (PFAS). Ace‐SABRE therefore may open up new opportunities for longitudinal studies of metabolic dysregulation and treatment efficacy without the need for a d‐DNP polarizer. At this stage in the development process, the optimized 35 mM pyruvate solutions delivered up to *p* = 17.3% polarization, corresponding to a molar polarization level of *p*_m_ = 6 mM. In contrast, the 70 mM pyruvate solutions yielded up to *p* = 12.5% polarization levels corresponding to a molar polarization of *p*_m_ = 9 mM. These polarization levels were reached by careful parameter optimization of SLIC *B*_1_ frequency, *B*_1_ amplitude, and catalyst concentration. It is noteworthy that catalyst optimization studies have delivered over 60% ^1^H and 80% ^15^N polarizations,^[^ ^75^, ^76^ ^]^ so we are confident that future developments will see this work produce outputs comparable to d‐DNP.^[^ ^1^, ^18^ ^]^

Subsequently, a processing protocol involving liquid–liquid extraction, gas stripping, and filtration was implemented to yield well‐tolerated injectables. Finally, HP spectroscopic tracking in a xenografted HCC tumor and HP MRI in healthy mice were demonstrated and compared to studies using d‐DNP as the polarization source. Clearly, Ace‐SABRE delivers sufficient polarization to facilitate rigorous analysis. However, at this stage in the development process, the images produced by d‐DNP provided better signal strength, we are confident that this difference will be overcome in the future. In summary, these advancements pave the way for the broader adoption of hyperpolarized imaging tools in preclinical research, with potential translation to clinical applications, representing a critical step toward fulfilling the promise of hyperpolarized MRI as a cornerstone technology in diagnostics and therapeutic monitoring.

## Supporting Information

The Matlab and Python codes used for data analysis can be made available upon reasonable request.

## Conflict of Interests

P.T. and C.D. are founders, employees, and equity holders of Vizma Life Sciences (hereafter, Vizma). M.S.R. and T.T. are also founders and equity holders of Vizma and serve on Vizma's scientific advisory board. E.Y.C. is an equity holder of Vizma and serves on Vizma's scientific advisory board. M.S.R. is a founder and equity holder of Hyperfine Inc. M.S.R. is an equity holder of DeepSpin GmbH. M.S.R. also serves on the scientific advisory boards of ABQMR, Synex Medical, Nanalysis, and O2M Technologies. E.Y.C. and B.M.G. are co‐founders and equity holders of XeUS Technologies Ltd.

## Supporting information



Supporting Information

## Data Availability

The data that support the findings of this study are available from the corresponding author upon reasonable request.
