# Supplementary material for: Scalable Hyperpolarized MRI Enabled by Ace‐SABRE of [1‐13C]Pyruvate
Source: Angew Chem Int Ed Engl. 2025 Jul 23;64(35):e202501231. doi: 10.1002/anie.202501231 (PMC12377446; doi:10.1002/anie.202501231)
Supplement: Supplementary file 1 — Supporting Information [file ANIE-64-e202501231-s001.docx]

Supporting Information for

Scalable Hyperpolarized MRI Enabled by Ace-SABRE of

[1-^13^C]Pyruvate

Stephen J. McBride^a+^, Megan Pike^a+^, Erica Curran^a+^, Alexander Zavriyev^b^, Bukola Abedesin^b^, Luke Tucker^c^, Jared M. Harzan^d^, Ishani M. Senanayake^d^, Mustapha Abdulmojeed^a^, Franziska Theiss^a^, Sheng Shen^e^, Thomas Boele^e,f^, Simon B. Duckett^j^, Boyd M. Goodson^d^, Matthew S. Rosen^e^, Eduard Y. Chekmenev^h^, Hong Yuan^g^, Carlos Dedesma^c^, Terence Gade^b^, Stephen Kadlecek^b^, Thomas Theis^*a,c,i^, Patrick TomHon^*c^

^a^ *Department of Chemistry, North Carolina State University, Raleigh, NC, USA*

^b^ *Department of Radiology, University of Pennsylvania, Philadelphia, PA, USA*

^c^ *Vizma Life Sciences, Chapel Hill, NC, USA*

^d^ *School of Chemical & Biomolecular Sciences, Southern Illinois University, Carbondale, IL, USA*

^e^ *A. A. Martinos Center for Biomedical Sciences, Massachusetts General Hospital, Charlestown, MA, USA*

^f^ *Image X Institute, Faculty of Medicine and Health, The University of Sydney, Sydney, NSW, AUS*

^g^ *Department of Radiology, University of North Carolina at Chapel Hill, Chapel Hill, NC, USA*

^h^ *Integrative Biosciences, Department of Chemistry, Karmanos Cancer Institute, Wayne State University, Detroit, MI, USA*

^i^ *Department of Physics, North Carolina State University, Raleigh, NC, USA*

^j^ *Department of Chemistry, University of York, York, UK*

**Abstract:** Hyperpolarized (HP) MRI using [1-13C]pyruvate is emerging as a promising molecular imaging approach. Among hyperpolarization methods, Signal Amplification By Reversible Exchange (SABRE) is attractive because SABRE polarizes the substrates directly in room-temperature solutions avoiding complex hardware. Most SABRE experiments have historically been performed in methanol, a relatively toxic and difficult to remove solvent. Here we demonstrate the use of a 80/20 acetone-water-solvent system (Ace-SABRE) to provide hyperpolarized [1-13C]pyruvate with up to 17 % polarization, then implement a solvent processing protocol to achieve injectable solutions retaining 74 % of the initial polarization, and lastly we demonstrate HP in vivo spectroscopy and imaging using the Ace-SABRE platform to showcase metabolic tracking in a hepatocellular carcinoma (HCC) tumor as well as HP-MRI, both in direct comparison to dissolution dynamic nuclear polarization (d-DNP) experiments. The Ace-SABRE technique promises faster adoption of SABRE hyperpolarization in biological experiments, overall lowering the barriers to entry for HP-NMR and HP-MRI.

**Table of Contents**

[**Experimental Procedures** 2](#_Toc193444847)

[Sample Preparation 2](#_Toc193444848)

[Experimental Setup and Hyperpolarization Experiments 2](#_Toc193444849)

[Purification and Processing of Hyperpolarized Samples 3](#_Toc193444850)

[In Vivo Experiments 3](#_Toc193444851)

[Polarization Quantification 4](#_Toc193444852)

[**Hyperpolarization of Different ^13^C Pyruvate Isotopomers** 5](#_Toc193444853)

[**Optimization of SLIC Hyperpolarization** 6](#_Toc193444854)

[**Analysis of Processed and Purified Hyperpolarized Pyruvate Solutions** 7](#_Toc193444855)

[Quantification of Excipient Solvents and Pyruvate Concentrations 7](#_Toc193444856)

[Quantification of Residual Iridium 10](#_Toc193444857)

[**Remaining Acetone, *n*-Butyl Acetate, and Iridium for Future Clinical Studies** 11](#_Toc193444858)

[**References** 12](#_Toc193444859)

### **Experimental Procedures**

## Sample Preparation

Parahydrogen was produced using enrichment at 25K using a commercially available parahydrogen generator (Advanced Research Systems, Inc.), yielding a parahydrogen fraction of ~95%. Experimental samples were prepared by mixing [1-^13^C] sodium pyruvate (Cambridge Isotopes Laboratories, 35 mM or 70 mM), DMSO-d6 (Cambridge Isotopes Laboratories, 12 mM or 24 mM), and organometallic SABRE catalyst (Ir-IMes, where IMes = 1,3-bis(2,4,6-trimethylphenyl)imidazol-2-ylidene)^[1]^ in 80% acetone (Sigma Aldrich HPLC grade) and 20% D_2_O (Cambridge Isotope Laboratories). Samples were sonicated for 30-60 seconds to dissolve all particles. All solvents were degassed with argon prior to sample formulation.

## Experimental Setup and Hyperpolarization Experiments

Samples were pressurized under 120 psi of parahydrogen gas in a 5 mm medium wall NMR tube (Wilmad Labglass). Samples were activated at 6.5ºC for 5-10 minutes of parahydrogen gas flow at a rate of 100 sccm through the solution. Samples were then polarized using 180s of parahydrogen gas flow at a rate of 100 sccm, a temperature of 6.5ºC, and a magnetic field of ~50 µT, while simultaneously applying a transverse magnetic field at the frequency of the ^13^C spins (~560 Hz). Details about the calibration of these spin-locked induced crossing (SLIC) pulses are given in the main text. Subsequently, an adiabatic pulse is applied with an amplitude of 1.5x the transverse pulse amplitude to transfer magnetization from the transverse to z-axis. This SLIC pulse scheme is used from prior work^[2,3]^. During this pulse the amplitude is decreased to 0 and the frequency of the B1 field is shifted to ±50 Hz from the ^13^C frequency (Figure S1). Note that all samples were polarized with a transverse (SLIC) pulse length of 180s except for the optimization experiments in Figure 2B and 2C, which were polarized for only 30s.

To detect the hyperpolarized ^13^C signal, samples were transferred under parahydrogen pressure into a 0.55 T benchtop MRI spectrometer (Pure Devices, GmbH).


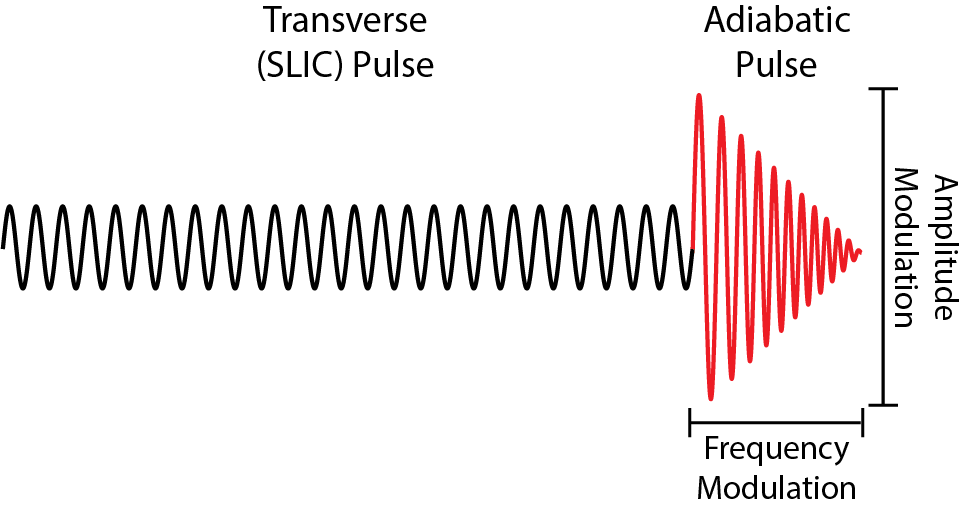


**Figure S1.** Schematic of the pulse sequence used for Ace-SABRE polarization (from SLIC SABRE).^[2–4]^ Amplitudes and durations are not shown to scale; the SLIC pulse is typically applied for 180 seconds and the adiabatic pulse is applied for 2 seconds.

## Purification and Processing of Hyperpolarized Samples

To prepare samples for measurement of residual excipients and *in vivo* experiments, additional processing steps are performed after hyperpolarization of the sample as above. After hyperpolarization of the pyruvate (180s bubbling with SLIC pulse and subsequent adiabatic pulse), the sample is removed from the shields and depressurized at a magnetic field of ~100 mT (the field is controlled at approximately ~100 mT for the entire processing protocol). The hyperpolarized sample in acetone and water is then mixed with *n*-butyl acetate. The resulting washed aqueous portion of the solution was inserted into a gas stripping apparatus, where nitrogen flow (>200 sccm) is applied to the solution at a temperature of >60ºC. The resulting processed solution is passed through a C18 column (Waters Sep-Pak Cartridge) to remove residual iridium in the sample. The resulting solution was diluted to 700 µL, buffered with Tris-EDTA buffer to a pH of 7.5 (40 mM Tris, 0.6 mM EDTA), and the osmolality was controlled to ~290 milliosmoles.

## In Vivo Experiments

The experimental procedures followed internationally accepted recommendations and guidelines for the handling of laboratory animals. All animal experiments were conducted at the University of Pennsylvania Perelman School of Medicine, and ethical approval for the animal experiments reported here was obtained from the relevant authority (U.S. Public Health Service, assurance number A3079-01). BALB/C and patient-derived xenograft hepatocellular flank tumor^[5]^ mouse models were used for the study. Anesthesia was induced using isofluorane and the animal’s vital signs were continuously monitored, adjusting the anesthesia as needed to maintain a respiration rate of approximately 70 breaths per minute. All necessary measures were taken to minimize the animals’ suffering and all animals were humanely euthanized after the experiments.

## Polarization Quantification


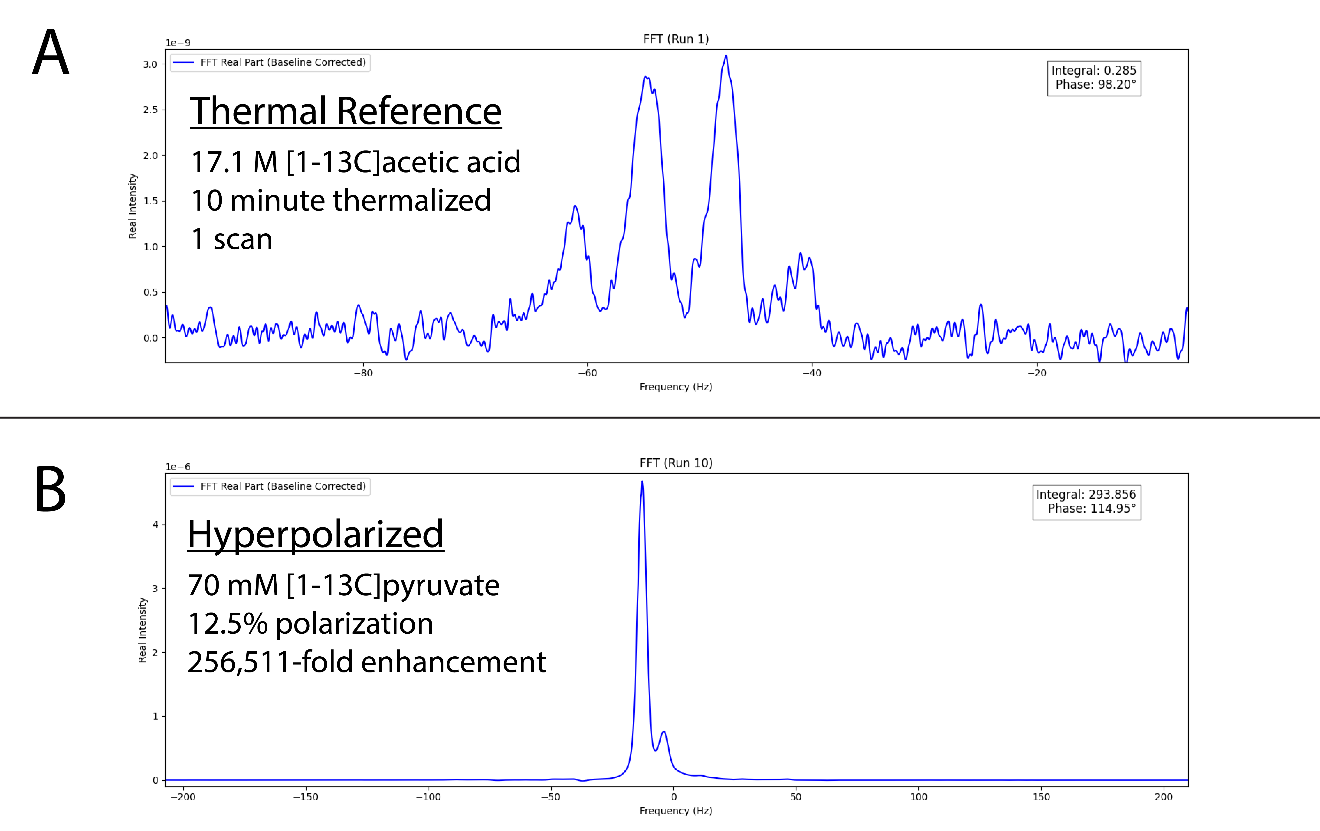


**Figure S2.** [A] Single ^13^C scan of pure [1-^13^C]acetic acid thermalized at 0.55 T and 303.15 K for ^13^C polarization calculations using the Pure Devices Benchtop MRI. [B] Example hyperpolarized spectrum of [1-13C]pyruvate measured in the 0.55 T Pure Devices Benchtop MRI.

A reference spectrum of pure [1-^13^C]acetic acid (**Fig. S2A**) was used to calculate ^13^C polarization for the Pure Devices Benchtop MRI (0.55 T), using the following function:

|  | $P=tanh\left( \frac{\gamma B_{0}\hbar}{2k_{B}T} \right)*\frac{S_{HP}}{S_{REF}}*\frac{C_{REF}}{C_{HP}}*100\%$ | **Eq. S1** |
| --- | --- | --- |

The tanh term calculates the thermal ^13^C polarization of the standard at a given magnetic field and temperature. The second term contains $S_{HP}$ and $S_{REF}$ which refer to the integrated signals of the hyperpolarized and reference samples. The third term contains $C_{REF}$ and $C_{HP}$ which refer to the concentrations of the reference sample (17.1M, [1-^13^C]acetic acid) and hyperpolarized samples, respectively. Hyperpolarized sample concentrations for [1-^13^C]pyruvate were either 35 mM or 70 mM for *in situ* samples. For purified samples, [1-^13^C]pyruvate concentrations were used as calculated above.

All integrals were calculated using Python code and all data was processed in either Python or Excel. Processing code and data are available on reasonable request.

### **Hyperpolarization of Different ^13^C Pyruvate Isotopomers**

**
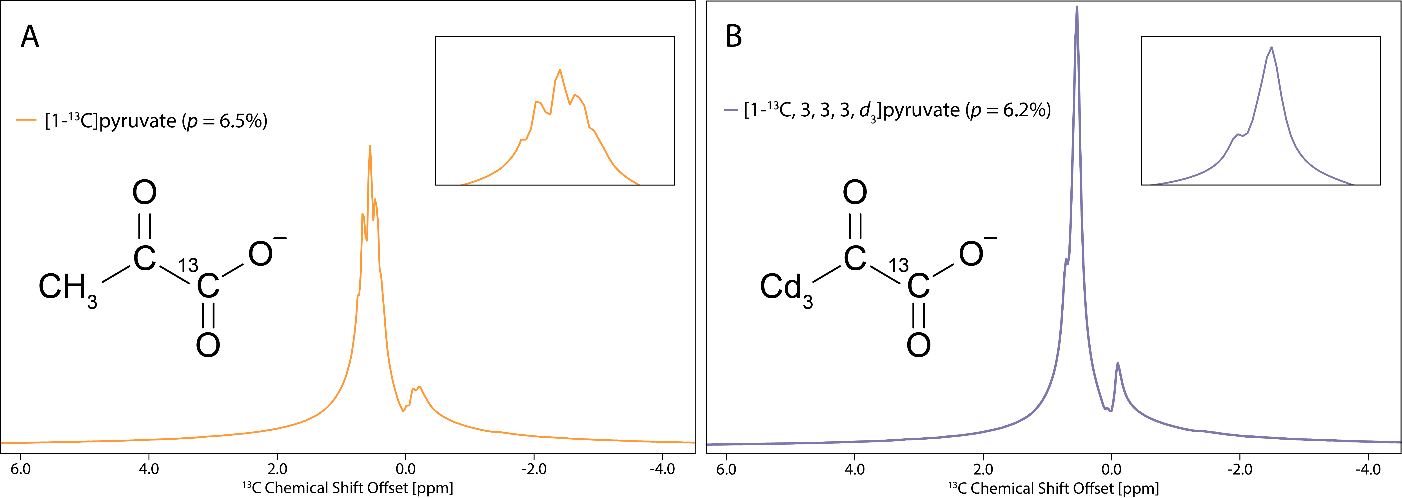
**

**Figure S3.** Overlayed ^13^C spectra of [1-^13^C]pyruvate (orange) and [1-^13^C, 3,3,3-d_3_]pyruvate (purple), both hyperpolarized using Ace-SABRE. Both samples use a 35 mM formulation of the respective pyruvate isotopomer with 12 mM DMSO and the respective 1:6 ratio of SABRE catalyst as described in the main text. Insets show the difference in observed splitting for both isotopomers, where the deuterated isotopomer does not have the couplings to the methyl group seen in the spectra of the non-deuterated molecule.

Previous applications of signal amplification using reversible exchange (SABRE) using spin-lock induced crossing and methanol as a solvent reported significant differences in polarization between the [1-^13^C, 3,3,3-*d*_3_]pyruvate (deuterated pyruvate) isotopomer in comparison to the fully protonated [1-^13^C]pyruvate species.^[6]^ We report that using the acetone-water systems employed in Ace-SABRE, ~6% polarization was observed for both the two pyruvate isotopomers (**Fig. S3**). The contrast between the results reported here and previously reported results^[6]^ are possibly due to differences in the dominant relaxation effects in different solvent environments and hence reaction networks. As referenced in the main text, these networks are known to be very complex and involve intermediates with the solvent molecules. In the previously reported methanol-based SABRE case the dipolar relaxation mechanism of the protonated or deuterated pyruvate methyl group coupling to the ^13^C spin is the dominant mechanism driving the difference between the two isotopomers. However, in these results we show that the solvent mixture used (acetone and water) mitigates the role of this mechanism to yield equivalent polarization with the two isotopomers, most likely due to differences in the chemical exchange of pyruvate (influencing the bound or free pyruvate relaxation) that will be explored in future work.

Notably, even at a detection field of 0.55 T (Pure Devices Benchtop MRI, 10 mm bore), we observe the expected differences in splitting of the ^13^C pyruvate signal between the two isotopomers (the [1-^13^C]pyruvate has an ~1 Hz quartet pattern, whereas the [1-^13^C, 3, 3, 3-*d*_3_]pyruvate spectra is a singlet).

### **Optimization of SLIC Hyperpolarization**

SLIC pulse parameters were optimized as described in the main text, sweeping the B1 power and frequencies to center the excitation on the spin transition necessary to drive SABRE polarization of [1-^13^C]pyruvate. For reproducibility, we provide below the data from Figure 2B and 2C in the main text:

**Table S1. SLIC B_1_ Frequency Optimization (as shown in Fig. 2B)**

| B_1_ Frequency (Hz) | Integral (a.u.) |
| --- | --- |
| 561 | -22.275 |
| 566 | -50.2 |
| 567 | -56.068 |
| 568 | -60.163 |
| 569 | -68.669 |
| 570 | -74.532 |
| 571 | -77.968 |
| 572 | -84.135 |
| 573 | -84.496 |
| 574 | -86.836 |
| 575 | -86.752 |
| 576 | 87.583 |
| 576 | -90.312 |
| 577 | 89.932 |
| 578 | 85.906 |
| 579 | 82.791 |
| 580 | 83.095 |
| 581 | 77.371 |
| 582 | 70.097 |
| 583 | 67.296 |
| 584 | 60.299 |
| 585 | 53.161 |
| 586 | 45.969 |
| 591 | 21.846 |

**Table S2. SLIC B_1_ Power Optimization (as shown in Fig. 2C)**

| B_1_ Power (microtesla) | Integral (a.u.) |
| --- | --- |
| 1 | 31.109 |
| 1.2 | 39.86 |
| 1.4 | 51.228 |
| 1.6 | 63.774 |
| 1.8 | 66.351 |
| 2 | 79.152 |
| 2.2 | 83.8 |
| 2.4 | 87.809 |
| 2.6 | 91.143 |
| 2.8 | 90.107 |
| 3 | 85.408 |
| 3.2 | 82.136 |
| 3.4 | 72.151 |
| 3.6 | 70.475 |
| 3.8 | 61.967 |
| 4 | 55.55 |
| 4.2 | 53.891 |

### **Analysis of Processed and Purified Hyperpolarized Pyruvate Solutions**

## Quantification of Excipient Solvents and Pyruvate Concentrations

Following the purification scheme outlined in **Fig. 1D** of the manuscript, the concentrations of acetone (Sigma Aldrich, HPLC grade >99.9%), *n-*butyl acetate (Sigma Aldrich, ACS reagent grade >99.5%), and [1-^13^C]pyruvate (Cambridge Isotope Laboratories, Inc.) remaining in the solution were quantified by NMR utilizing a calibration curve. The calibration curve for each compound was prepared via serial dilution of each compound. 500 µL of 99.9% D_2_O (Cambridge Isotope Laboratories, Inc.) was added to 500 µL of each calibration curve standard solution, and triplicate standards at each concentration were measured using a 400 MHz Bruker NMR. Spectra for each sample (^1^H for acetone and *n-*butyl acetate, ^13^C for pyruvate) were obtained in triplicate. The ^1^H chemical shifts corresponding to acetone and *n-*butyl acetate integrals were 2.01 ppm and 0.69 ppm, respectively, while the chemical shift for [1-^13^C]pyruvate was 170 ppm. The average integrals and standard deviations are presented in **Table S3** (acetone), **Table S4** (*n-*butyl acetate), and **Table S5** (pyruvate). The resulting calibration curves are presented in **Fig. S4** (acetone), **Fig. S5** (*n-*butyl acetate), and **Fig. S6** (pyruvate).


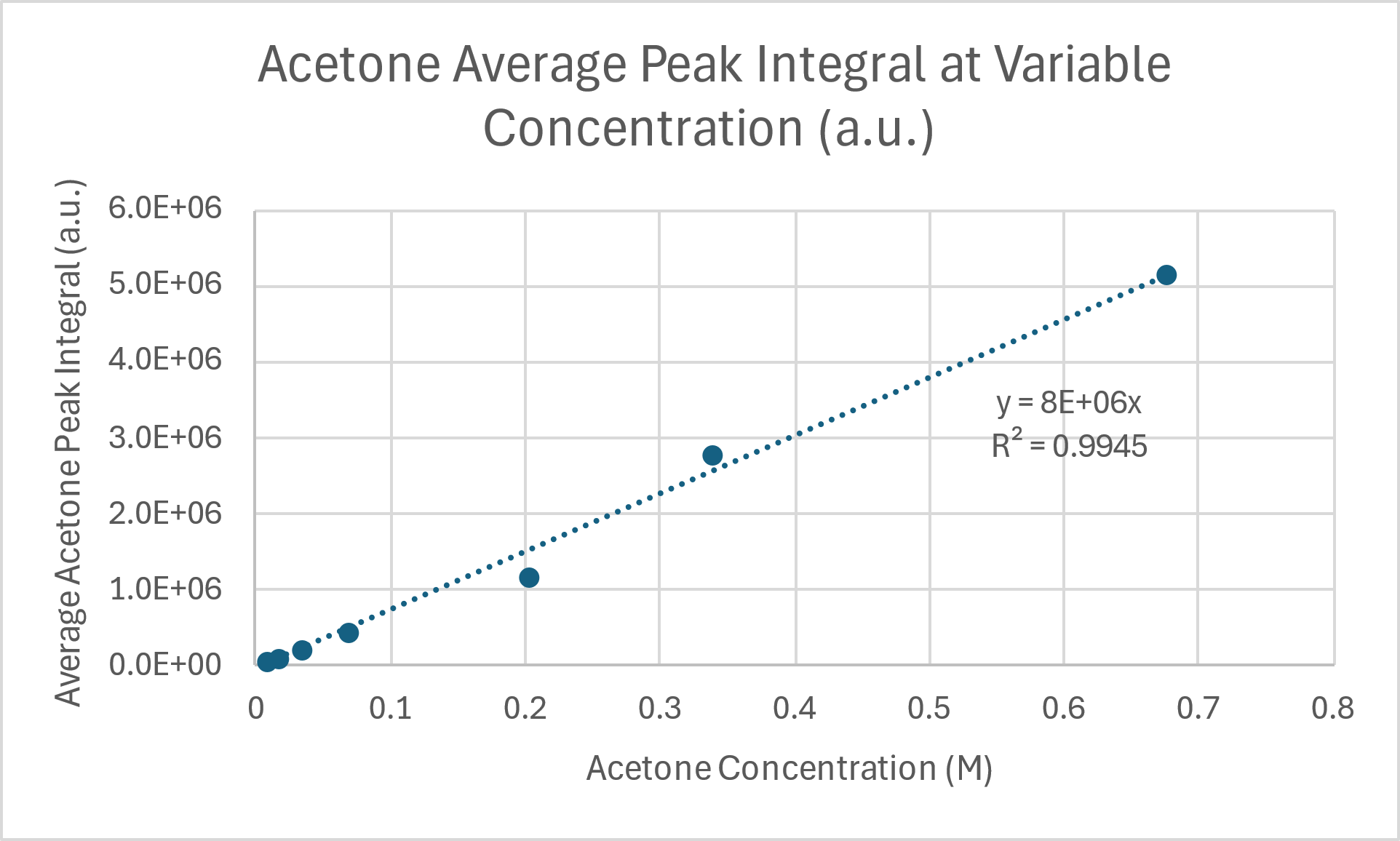


**Figure S4.** Average acetone ^1^H integral (n=3) at variable concentration.

**Table S3. Acetone ^1^H Integral at Variable Concentrations**

| **Acetone Concentration (mM)** | **Acetone Average Peak Integral (a.u.)** | **Acetone Peak Integral SD (a.u.)** |
| --- | --- | --- |
| 675.0 | 5151396 | ± 250922 |
| 337.5 | 2767405 | ± 125904 |
| 202.5 | 1152827 | ± 139373 |
| 67.5 | 447077 | ± 41421 |
| 33.7 | 209703 | ± 18964 |
| 16.9 | 107378 | ± 8714 |
| 6.7 | 42197 | ± 769 |


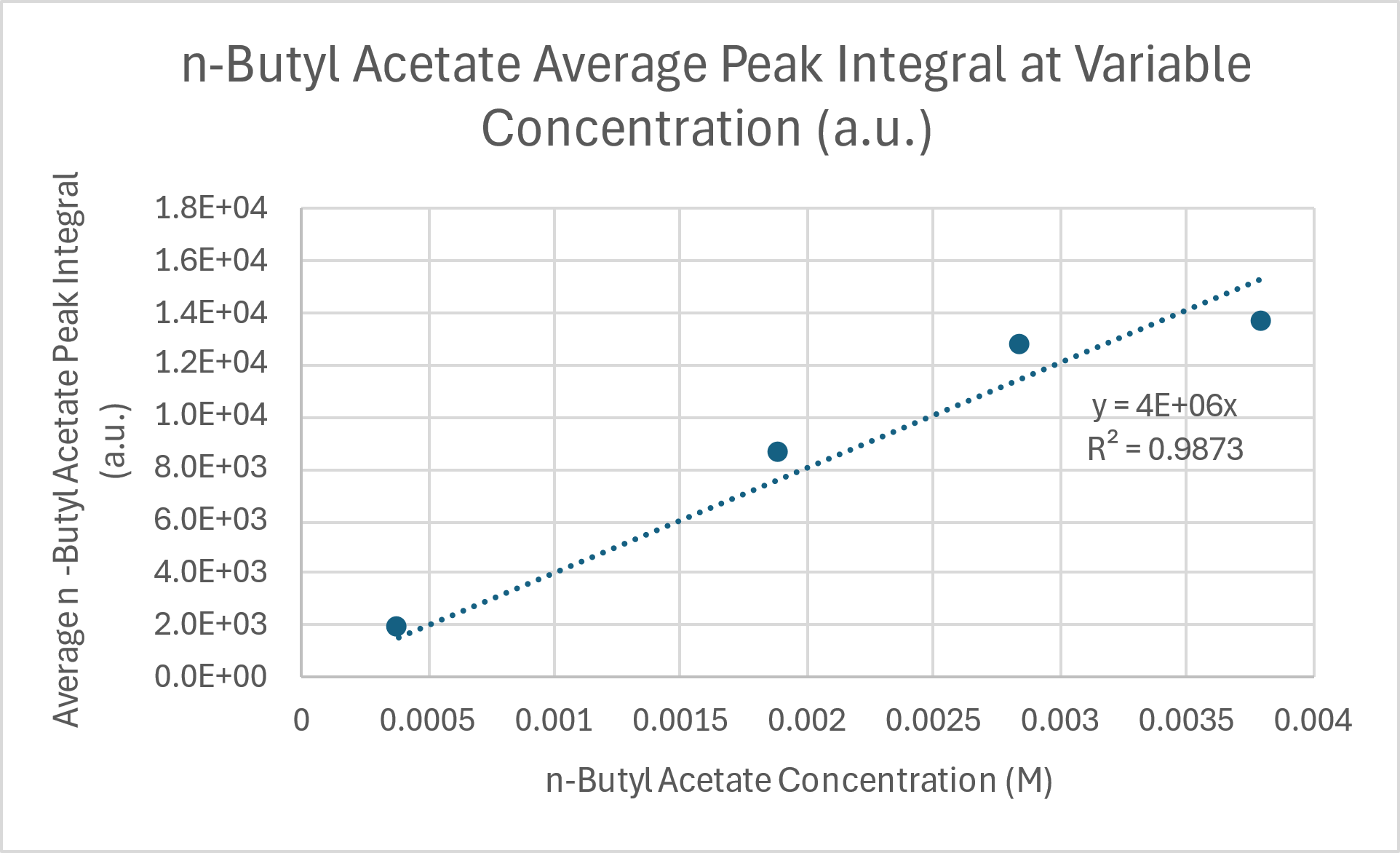


**Figure S5.** Average n-butyl acetate ^1^H integral (n=3) at variable concentrations.

**Table S4. *n*-Butyl Acetate ^1^H Integral at Variable Concentrations**

| ***n*-Butyl Acetate Concentration (mM)** | ***n*-Butyl Acetate Average Peak Integral (a.u.)** | ***n*-Butyl Acetate Peak Integral SD (a.u.)** |
| --- | --- | --- |
| 4 | 13690 | ± 147 |
| 3 | 12787 | ± 394 |
| 2 | 8592 | ± 72 |
| 0.4 | 1939 | ± 111 |


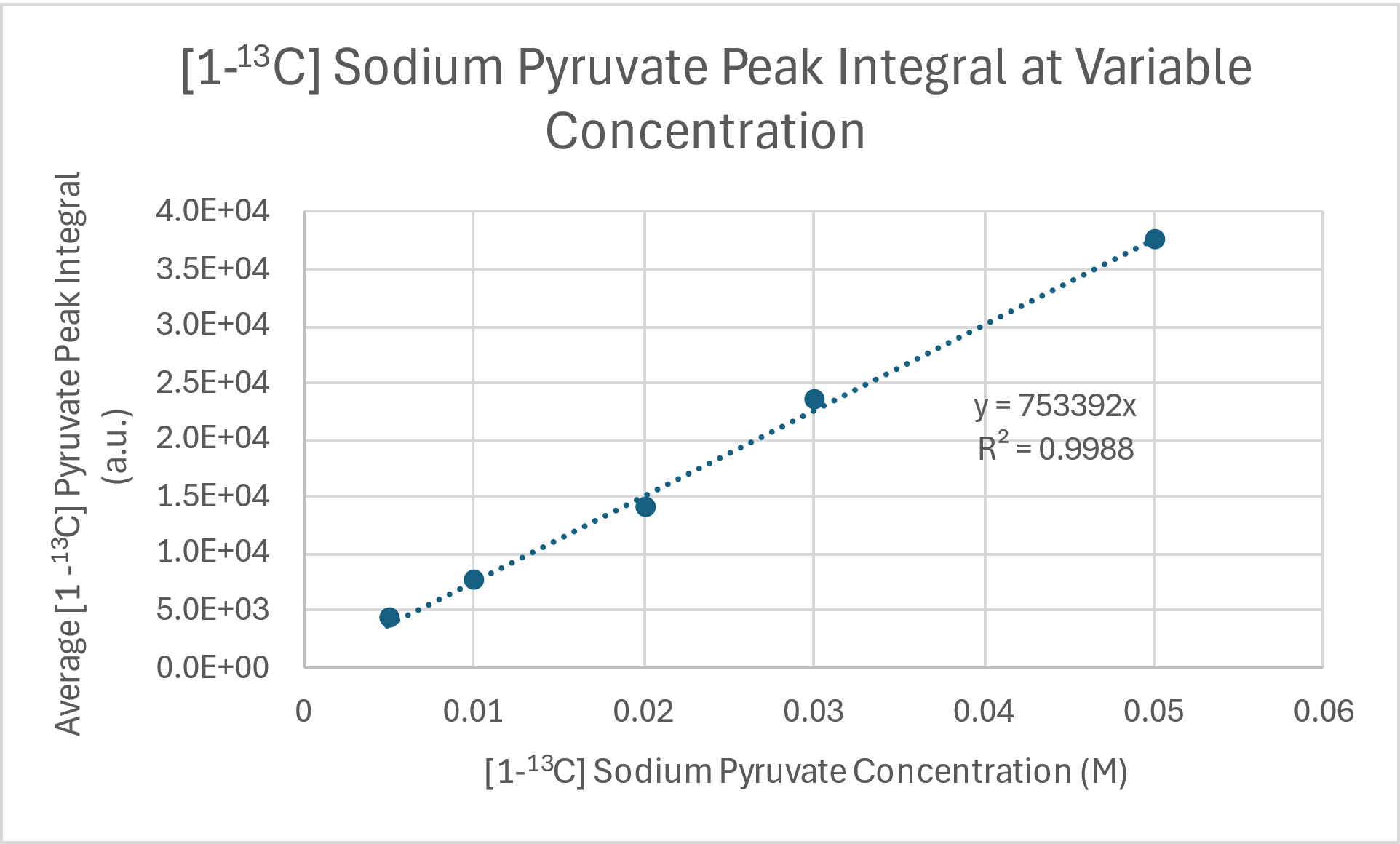


**Figure S6.** Average [1-^13^C]pyruvate ^13^C integral (n=3) at variable concentrations.

**Table S5. [1-^13^C]pyruvate ^13^C Integral at Variable Concentrations**

| **[1-^13^C]Pyruvate Concentration (M)** | **Average [1-^13^C]Pyruvate Peak (a.u.)** | **Standard Deviation Acetone Peak (a.u.)** |
| --- | --- | --- |
| 50 | 37461 | ± 7437 |
| 30 | 23539 | ± 2771 |
| 20 | 13945 | ± 1160 |
| 10 | 7656 | ± 3390 |
| 5 | 4479 | ± 2295 |

The resulting calibration curves are calculated via a linear fit with *b* set to 0, with the equations shown below: acetone (ace, **Eq. S2**), *n*-butyl acetate (BA, **Eq. S3**), and pyruvate (pyv, **Eq. S4**); where *PI* is the average peak integral, and *c* is the concentration.

${PI}_{ace}={7597712 \times c}_{ace}$ Eq. S2

${PI}_{BA}={4022669 \times c}_{BA}$ Eq. S3

${PI}_{pyv}={753392 \times c}_{pyv}$ Eq. S4

Residual solvent excipient concentrations in purified samples were quantified using the calibration curves above. 500 µL of each purified sample was diluted with 500 µL of 99.9% D_2_O (Cambridge Isotope Laboratories, Inc.). NMR spectra were obtained in triplicate using a 400 MHz Bruker and the resulting acetone, *n*-butyl acetate, and pyruvate concentrations are presented in **Table S6** (also shown in Table 1 in the main text).

**Table S6. Excipient and Pyruvate Concentrations for Purified Samples**

| **Sample** | **Acetone Concentration (mM)** | ***n*-Butyl Acetate Concentration (mM)** | **[1-^13^C]Pyruvate Concentration (mM)** |
| --- | --- | --- | --- |
| Sample 1 | 97.4 | 0.7 | 65 |
| Sample 2 | 154.6 | 0.7 | 70 |
| Sample 3 | 107.9 | 0.6 | 65 |
| Average | 120.0 ± 25 | 0.7 ± 0.1 | 67 ± 2 |

## Quantification of Residual Iridium

While the processing described (liquid-liquid extraction and subsequent filtration) removes most of the iridium in the solution, a small amount remains. Following the purification scheme outlined in **Fig. 1D** of the manuscript, residual iridium concentration was determined using Inductively Coupled Plasma Mass Spectroscopy (ICP-MS). Each sample was diluted into two different concentrations (1:1 dilution and a 1:10 dilution) using 2% nitric acid. The purified samples and a blank were analyzed and the resulting Ir concentrations are presented in **Table S7**.

**Table S7. Iridium Concentrations for Purified Samples Calculated Using ICP-MS**

| **Sample** | **Ir Concentration** |
| --- | --- |
| Sample 1 | 2.2 mg L^-1^ |
| Sample 2 | 3.9 mg L^-1^ |
| Sample 3 | 2.0 mg L^-1^ |
| Average | 2.7 ± 0.8 mg L^-1^ |
| Blank | 5.0 µg L^-1^ |

### **Remaining Acetone, *n*-Butyl Acetate, and Iridium for Future Clinical Studies**

The levels of excipients reached in this study demonstrate the viability of the Ace-SABRE technique for preparation of pre-clinical grade hyperpolarized pyruvate and show feasibility of preparation of near-clinical levels of the remaining organic (acetone, *n-*butyl acetate) and metal (iridium) excipients. **Table S8** below shows the levels of excipients achieved in this work relative to the FDA/ICH guidelines for the permissible daily exposure (PDE) for each excipient.^[7,8]^ The PDE is the level of daily exposure to a compound a person can experience at or below which no adverse effects are expected to occur.

**Table S8. FDA/ICH PDE levels and current excipient concentrations achieved with Ace-SABRE**

| Excipient | Ace-SABRE Processed Concentration | PDE Concentration (for 50 mL dose) |
| --- | --- | --- |
| Iridium (µM) | 14 ± 4 | 1.0 |
| Acetone (mM) | 120 ± 25 | 72.3 |
| *n*-Butyl Acetate (mM) | 0.7 ± 0.1 | 14.3 |

PDE values given in mg/day have been converted to concentrations relative to a standard 50 mL clinical dose of hyperpolarized pyruvate. Notably, Ace-SABRE and the processing methods described achieve compatibility with the *n*-butyl acetate PDE concentration and are only 14-fold, and 1.6-fold away from the iridium and acetone PDE thresholds. Additionally, we note that the PDE thresholds are established to “be protective of public health for all patient populations” and that the FDA/ICH additionally describes that “levels of impurities higher than an established PDE may be acceptable in certain cases.”^[8]^ These cases include intermittent dosing, short term dosing (<30 days), and specific indications.^[8]^

### **References**

[1] B. J. Tickner, O. Semenova, W. Iali, P. J. Rayner, A. C. Whitwood, S. B. Duckett, *Catal Sci Technol* **2020**, *10*, 1343–1355.

[2] S. J. Devience, R. L. Walsworth, M. S. Rosen, *Phys Rev Lett* **2013**, *111*, DOI 10.1103/PHYSREVLETT.111.173002/FIGURES/3/THUMBNAIL.

[3] A. B. Schmidt, J. Eills, L. Dagys, M. Gierse, M. Keim, S. Lucas, M. Bock, I. Schwartz, M. Zaitsev, E. Y. Chekmenev, S. Knecht, *Journal of Physical Chemistry Letters* **2023**, *14*, 5305–5309.

[4] A. N. Pravdivtsev, I. V. Skovpin, A. I. Svyatova, N. V. Chukanov, L. M. Kovtunova, V. I. Bukhtiyarov, E. Y. Chekmenev, K. V. Kovtunov, I. V. Koptyug, J. B. Hövener, *Journal of Physical Chemistry A* **2018**, *122*, 9107–9114.

[5] N. R. Perkons, R. M. Kiefer, M. C. Noji, M. Pourfathi, D. Ackerman, S. Siddiqui, D. Tischfield, E. Profka, O. Johnson, S. Pickup, A. Mancuso, A. Pantel, M. R. Denburg, G. J. Nadolski, S. J. Hunt, E. E. Furth, S. Kadlecek, T. P. F. Gade, *Hepatology* **2020**, *72*, 140–154.

[6] H. de Maissin, P. R. Groß, O. Mohiuddin, M. Weigt, L. Nagel, M. Herzog, Z. Wang, R. Willing, W. Reichardt, M. Pichotka, L. Heß, T. Reinheckel, H. J. Jessen, R. Zeiser, M. Bock, D. von Elverfeldt, M. Zaitsev, S. Korchak, S. Glöggler, J.-B. Hövener, E. Y. Chekmenev, F. Schilling, S. Knecht, A. B. Schmidt, *Angewandte Chemie International Edition* **2023**, *62*, e202306654.

[7] “Q3C(R8) Impurities: Guidance for Residual Solvents Guidance for Industry | FDA,” can be found under https://www.fda.gov/regulatory-information/search-fda-guidance-documents/q3cr8-impurities-guidance-residual-solvents-guidance-industry, **n.d.**

[8] “Q3D(R2) – Guideline for Elemental Impurities | FDA,” can be found under https://www.fda.gov/regulatory-information/search-fda-guidance-documents/q3dr2-guideline-elemental-impurities, **n.d.**
